# Physical Condition of the Aborigines—with an Account of Their Practice of Medicine

**Published:** 1848-01

**Authors:** 


### ECLECTIC DEPARTMENT,

### AND SPIRIT OF THE MEDICAL PERIODICAL PRESS.

---

*Physical Condition of the Aborigines—with an account of their Practice of Medicine.* By EDWIN G. MEEK, M. D., of Choctaw Agency, West of Arkansas.

To one actually conversant with the details of Indian customs and modes of life, it is singular how vague and incorrect are the notions of even citizens of the United States concerning the aboriginal inhabitants of this country.

Much that has been said on this subject, is about as worthy of belief, as the narratives of Captain Guliver and Baron Munchausen. By many who profess to be admirers of nature and primitive simplicity, the Red Man has been cited to prove the inutility of the medical and surgical theory and practice of the whites, and to prove that beyond the employment of a few simple vegetable remedies, the Science of Medicine is rather a curse than a blessing to the human family.

At this age of the world it is scarcely necessary to meet such absurdities with even a formal denial; but perhaps the actual state of disease and the healing art among this simple people, might be a source of gratification to some of the numerous readers of your magazine.

There is one difficulty connected with this subject: many of the tribes have held more or less intercourse with the whites, and some of their medical notions have been thereby somewhat modified. The writer purposes in this article, to give such facts only as have come to his knowledge, during a residence of more than two years amongst the Choctaws, west of the Mississippi.

The country inhabited by this tribe, lies between about 34° and 25° 40' N. latitude, and between 18° and 20° longitude west of Washington, having the Arkansas river on the North, and the Red River on the South; the larger portion of the tribe being located near the later stream. The climate is as salubrious as any other in the same latitude elsewhere; the winters are mild though variable, and the summers are not usually oppressively hot. The principal diseases are Pneumonia, Pleuritis, Intermittent and Bilious Remittent fever, Enteritis and occasionally Scrofula. Gonorrhœa and Syphilis are of occasional occurrence, though not so frequent as amongst the other tribes. There is no reason to believe that venereal diseases were known to any of the tribes, before they had intercourse with the Europeans, nor that they are generally disposed to licentiousness; such diseases are known among them by the very appropriate designation of *Lu-ak* (fire).

The *Ah-lek-che* or Medicine man is generally a pretty shrewd, cunning character, and sometimes manages to obtain considerable influence with a certain class, although there is a large number who treat his pretensions with contempt; indeed it may be safely affirmed, that a majority of the nation prefer to receive the attention of a white physician, when one can be obtained. These conjurors, have of course, no correct knowledge of Anatomy, Physiology, or Pathology; they ascribe all diseases to the malignant influences of wizards, witches and evil spirits, and occasionally point out an old man or woman to whom supernatural influences of this nature is attributed: of course these unfortunate beings are the objects of hatred, and so bitter is the animosity entertained against them, that the Council has enacted a statute, making it a penal offence, punishable by severe whipping, to accuse one of witchcraft.

When the doctor is called to his patient, he commences operations by excluding all white men, and all who disbelieve in the efficacy of his incantations: he then commences a prodigious shaking of a gourd containing some dry beans, peas, pebbles or something of that nature, accompanying the process with frantic howlings, and violent gesticulations, the object of which is to frighten away the evil spirit. Should there be violent local pain, as in pleurisy, he sucks the affected part, and not unfrequently shows a piece of cane root, or something of the sort, which he professes to have extracted from the patient, and which he declares had been shot into the sufferer by a witch, thus causing the disease by which he was afflicted. In all acute affections the above is deemed the most efficacious mode of treatment; should it fail to produce the desired effect, the conjuror ingeniously excuses his want of success by averring that the witch or wizzard who primarily induced the disease, is in the company, and by informal machinations reproduces the complaint as fast as he cures it. Keen and fiery are the glances that are then cast over the company by the friends of the patient—and wo to the toothless old man, or withered crone upon whom suspicion alights; in the excitement of the occasion, the infuriated company would not hesitate for a moment to take the life of any one whom their judgment convicted of the nefarious practice of witchcraft; fortunately for such, the statute before mentioned maintains a salutary restraint on the tongue of the conjuror.

In addition to such means, they use as adjuvants a few simple vegetable remedies, vapor baths made from cedar twigs, decoction of *Eupatorium Perfoliatum*, and divers things of the like nature. I have not been able to learn whether or not venesection was a common therapeutic agent amongst them prior to their communication with the whites; it is a means of cure to which the common Indians are very partial, though the conjurors view it with jealousy, deeming it rather an innovation upon their practice.

As may be inferred, inflammatory diseases, and especially those of the lungs and pleural, are fatal amongst them; in fact these diseases are peculiarly terrible, especially when they prevail epidemically: during the last winter, Typhoid Pneumonia prevailed extensively and was attended with frightful mortality. Singular as it may seem, to residents of a higher latitude, I have no hesitation in ascribing the frequency of pulmonary affections, in good part, to the mildness of the winter: there is seldom more than four or five days in succession of very cold weather, and the consequence is that the inhabitants do not take the precaution to protect themselves sufficiently against the vicissitudes of the season. There are however, other causes to be taken into the account; not unfrequently the thermometer will indicate a variation of twenty degrees in two or three hours; in fact we have often a specimen of the four seasons in the course of twenty-four hours; to these great and sudden changes they take but little care to adapt their clothing, and the result is what might be reasonably anticipated. Another cause which may be mentioned as a prolific source of these affections, is the great exposure to which the Indians subject themselves, especially during a drunken frolic, when they will sometimes lie the entire night exposed to a rain or snow storm with no other clothing than a shirt and leggins. There is no doubt that acute inflammatory affections are more amenable to proper treatment, amongst the Indians than amongst the Whites; the former seem to bear depletion much better than the latter: the effect seem to follow more the use of the remedy, and the impression is more decided and permanent. This may be owing to the fact that their habits of life are more natural and simple than ours: whatever may be the cause, there is no doubt of the fact. I regard it as well settled that a timely use of the lancet and Tatar Emetic will amongst them cure Sthenic Pneumonia, as that Quinine will check an Intermittent. Their fevers are generally easily managed, and are less fatal than the same diseases are amongst us: in fact it is rarely that they die with an idiopathic fever. The great difficulty a physician has to encounter, is the want of judicious nursing and proper diet: being in this, as in other things, practical fatalists, they regard an attack of disease inevitable, and treat it with indifference.

In chronic complaints, the native physicians are of course even less successful than in acute diseases. Here the resources of their Materia Medica are totally inadequate to the expulsion of the evil spirit, or the counteraction of the wizard's spell. There is no authentic account that they ever had any knowledge of an oxide, or any form of metallic remedies, and the grand arsenal of weapons which Chemistry has furnished the scientific physician, has never been opened to the use of the Red Man. In the treatment of Scrofula, Consumption, or any diseases that require skill in the selection, or judgment in the application of remedies, the Indian Doctor is totally in the dark, and unless the white man can relieve his sufferings, the poor invalid lingers on, looking hopefully to death as the termination of his sorrows.

Their Surgery like their Medicine, is very rude and unskilful. It has been asserted that they have remarkable success in the treatment of gunshot wounds, cuts stabs; and so general is the belief, that the War Department in a set of queries propounded to those supposed to be familiar with Indian affairs, assumes the fact as established, and requests to know whether it is the "result of the particular mode of treatment, or the assiduity and care of the physicians." Now, as far as my observation and experience go, there is no reason to believe that they possess any remarkable skill, or that their success is such as it has been sometimes represented to be. I have attended many who were badly wounded in their numerous drunken affrays, and have noticed many unseemly cicatrices, the results of former wounds, and such as any surgeon of reasonable pretensions would be ashamed to show as the consequences of his treatment of incised wounds. The native surgeons are totally unskilled in the use of stitches, sutures and adhesive plasters, so that a severe wound has no opportunity of healing by the first intention; neither have they the necessary art of properly applying a roller, that *sine qua non* of a surgeon's apparatus. In fact their treatment of such wounds amounts to nothing, or sometimes worse; and the success attributed to them belongs properly to nature alone. It is somewhat singular that although they use in their fights, unsatisfactory looking butcher's knives, they rarely kill each other. If two white men were to fight with such weapons, the great probability is that one or both would be killed, but the Indian uses his knife to slash and hack, not usually aiming to stab, But one instance of a fatal injury being inflicted in this way has ever come to my notice: in that instance the knife had penetrated the lung. I did not see the patient until the third day after the wound was inflicted, and suppuration and effusion had then commenced: had proper treatment been instituted sufficiently early, the chances were that the man would have recovered. They do not often sever important arteries, and so little are they acquainted with anatomy, that such wounds are peculiarly dangerous, when the assistance of a competent surgeon cannot be secured. To the fact that their wounds are not generally dangerous, is to be, in great part, imputed their skilful treatment. When a really dangerous wound is received, the result is usually fatal, unless better means of cure can be obtained than the Indian possesses: The same remark may be made in reference to gun-shot wounds: their instruments for the extraction of a ball are rude and clumsy, and if the ball be difficult to come at, they fail to extract it. Their treatment of contusions, sprains, luxations and and fractures, is more skilful than any other part of their surgical performances. Luxations and fractures frequently occur at their ball plays, (which are sports of the most violent character) and are generally reduced on the spot, and so well that deformity rarely, if ever, results; but the contrivances they use are clumsy and ill-formed. I have never known of their performing amputation in any case; and do not suppose they have the necessary knowledge and skill in the management of severed arteries, to say nothing of proper knives, tourniquets, adhesive plasters, lints and bandages.

With regard to surgical diseases, their treatment is as well adapted to produce a cure, as that used for the inflammatory affections before mentioned. Ulcers are very frequently met with and are peculiarly obstinate: they result from the same causes that produce them elsewhere, filthiness, sequela of fever, insufficient or innutritious diet, improperly treated wounds, or a generally cachectic condition of the system. The natives regard them as incurable; but a proper application of bandages and lotions will often produce a favorable result. Carbuncles, biles, abscesses, and other external inflammations, are generally allowed to run their course without interruption.

There are here various noxious reptiles and insects whose bite is poisonous; the large and the ground Rattlesnake, the Moccason and the Cotton mouth snakes, the Scorpion, Centipede and Tarantula, The bite of none of these is usually fatal, or do I know that the natives possess any remedies that may be regarded as specifics in such cases. There is reason to believe that the noxious qualities of these *varmints* have been somewhat overrated, although they are in sober verity, dangerous looking things, especially the Tarantula, concerning which the same notion exists that is recorded in Tweedie's Library, that its bite is curable by lively music. I once proposed to try the effect of this agent upon an Indian, but he very emphatically declined the risk; preferring a whole skin to a poisoned wound, with nothing but "Yankee Doodle," or "Old Dan Tucker" for Medicine, *Aqua Ammonia* would generally be efficacious in such cases, but like the before mentioned individual, I have never had sufficient curiosity to induce me to try the experiment upon myself.

Concerning Obstetrics amongst them, there is not much to be said. As a general thing, child-birth is not characterized by much risk of suffering: accidents and protracted labors are very rare. The treatment of the latter is rude and barbarous to the last degree: the attendants resort to kneading the abdomen with the fists, and this failing, they get on the patient with their knees and use great violence. This branch of the profession is left entirely to women, and the cases are very rare in which a physician is resorted to. They use different postures in delivery, perhaps the sitting posture is as common as any other. It is perhaps not neces- to state the reasons why labors are so easy amongst them: every physician is familiar with the general influence of civilization upon the female constitution: I will, however, venture to suggest the indifference to pain, and the stoicism of the Indians are partly attributable to a less delicately organized nervous system than the white race possess. Female diseases are not so common amongst them, as with the whites; and owing to their diffidence in such matters, they do not often consult a physician in such cases.

The treatment of children is not such as is generally said to prevail amongst the Aborigines: the children are not bound to a board, but dressed after the custom of the whites. They do not receive proper care and attention, and the mortality amongst them is very great. Emigration and change of residence also prove very injurious to children. A great proportion of the deaths amongst those who have migrated from the East bank of the Mississippi, to their new homes in the West, has been amongst the children: next to these the aged suffered most.

Comparisons have been often instituted between the white and red races, with reference to their comparative longevity, capacity for sustaining fatigue, muscular strength, &c.: and here again the white skin has usually the advantage. The Indians very rarely attain a great age; even those who possess originally good constitutions, and live peaceful, quiet lives. It would seem to be a law of nature, that the mental, equally with bodily organization, needs a due degree of exercise, and this law the Indian habitually transgresses. Their mental faculties are employed only at irregular intervals, and then the feelings more than the intellect: there is not that steady, equable excitement which civilization affords to our race. The Indian's intellect becomes torpid, and there can be no doubt that his indolence shortens his days. The average duration of a generation with them is less than with us: from the best data I have been able to obtain, the average length of their term of life does not exceed seventeen years at the farthest. In the year 1837, a party of five hundred Choctaws volunteered to go to Florida to fight the Seminoles: they were not however mustered into service: these men were either young or in the prime of life; and in looking over their muster roll, it is ascertained that more than half of them are now dead. When we add that there is a smaller proportion of births amongst them than with us, we cannot wonder that they are fast fading away from the earth, and that before long they will be reckoned among the nations that have gone forever. A melancholy chapter might be written on the agency of civilization in producing this state of affairs, but this is not the place for such a discussion.

Their muscular strength and capacity of bodily endurance, like many other things with them, are matters of impulse; but when it comes to steady endurance for a length of time they are found wanting. It would be unreasonable to suppose, that with their habits of life, they would be equal in this particular to a laboring white man. They will sustain for a short time the most violent efforts, in the chase or at a ball play, for which they compensate by days of indolence and inactivity. So the wild tribes of the prairies will exist days upon parched corn, and at the end of their fast will devour ten pounds of meat in a day: this may startle some of your readers, but the records of the War Office will tell you it is no exaggeration.

It has been said that an Indian never commits suicide: this is a mistake: I have known instances of it amongst the Choctaws, and once at least where no cause was known for it. If insanity occurs amongst them, I have never seen or heard of it.

I have thus in a desultory form thrown together such matters as came first concerning the physical condition of this interesting people: if your readers be thereby gratified, the writer will be amply repaid; if not, he will have the satisfaction of knowing that he is not the first who has turned blank paper to profitless uses.

*September*, 1847. [*Ill. and Ia. Med. and Surg. Journal.*

